# Norovirus infections in preterm infants: wide variety of clinical courses

**DOI:** 10.1186/1756-0500-2-96

**Published:** 2009-06-02

**Authors:** Sven Armbrust, Axel Kramer, Dirk Olbertz, Kathrin Zimmermann, Christoph Fusch

**Affiliations:** 1 Department of Neonatology, University-Children's Hospital, 17475 Greifswald, Germany; 2 Institute for Hygiene and Environmental Medicine, Ernst Moritz Arndt University Greifswald, 17475 Greifswald, Germany; 3 Department of Neonatology, Suedstadt Clinic, 18059 Rostock, Germany; 4 Friedrich Loeffler Institute for Medical Microbiology, Ernst Moritz Arndt University Greifswald, 17475 Greifswald, Germany

## Abstract

**Background:**

Norovirus is an important cause of nonbacterial acute gastroenteritis in all ages. Atypical courses are described. Clinical symptoms are diarrhea, vomiting, nausea, abdominal cramps, fever and malaise. Apart from three recent short reports we describe for the first time an outbreak of norovirus in a tertiary Neonatal Intensive Care Unit.

**Findings:**

The typical symptoms of norovirus infection are in part also seen in premature born infants but with a different pattern and a huge variety of clinical courses. Vomiting is not the main symptom of norovirus infection in premature infants but distended abdomen and other symptoms such as apnea, gastric remainders or sepsis like appearance. The course in premature born patients could be explained by an immunocompromised mice model. Extensive hygienic measures were necessary to control the outbreak without closing the Neonatal Intensive Care Unit.

**Conclusion:**

Norovirus infection in premature infants shows an impressive pattern of a wide variety of clinical courses. Only the consequent use of different hygienic pattern can lead to elimination of norovirus.

## Background

Norovirus, belonging to the family of Caliciviridae, is a highly contagious virus and has been found to be one of the most important causes of nonbacterial acute gastroenteritis in all ages in developing as well as in developed countries [1-3]. While most of the outbreaks are known to have a seasonal pattern, sporadic cases of disease throughout the year are described [4,5]. Although outbreaks can occur in a variety of settings, semiclosed communities like hospitals are favoured [6]. Norovirus are transmitted through common sources such as food and water, person-to-person contact or airborne via aerosolized vomit whereas an extremely small dose of virus patricles (3 – 10) already can lead to infection [7-9]. Norovirus can be detected by ELISA as in this report, by RT-PCR or electron microscopy.

A lot of reports and studies about norovirus and its outbreaks exist covering a wide range of different areas and age groups with the few, typical clinical symptoms. Atypical courses of the disease are described in immunocompromised patients and persons under severe stress [10,11].

Cause of this outbreak was a mother of a hospitalised preterm baby. She suffered from typical clinical signs of norovirus infection including acute diarrheal illness and was tested positive for norovirus shortly before the onset of clinical symptoms in our patients.

Apart from three recent reports dealing with norovirus outbreaks in premature infants we describe for the first time an outbreak of norovirus infections, its clinical course and control with extensive hygienic measures in a tertiary Neonatal Intensive Care Unit (NICU) affecting 11 infants over a period of two months showing an impressive pattern of a wide variety of clinical courses [12,13].

## Case Report

### Case 1

26 weeks of gestation, 760 g, on nasal CPAP, on day 14 acute severe worsening of the general condition, anemia, increasing oxygen demand, sepsis-like clinical appearance with increasing apnea followed by intubation and ventilation for 7 days, proof of norovirus for three weeks also in tracheal aspirate.

### Case 2

26 weeks of gestation, 820 g, initial clinical course without complications, on day 34 sudden development of tachycardia, restlessness, distended abdomen and worsening of general condition, sepsis-like clinical appearance with tachypnea, thrombocytopenia and anemia, increasing oxygen demand. Proof of norovirus, later also rota- and astrovirus. Child developed necrotizing enterocolitis 2B° and underwent surgery with left sided hemicolectomy and ileostoma.

### Case 3

29 weeks of gestation, 1150 g, on day 5 acute worsening of the general condition, increasing gastric remainders, vomiting, distended abdomen, proof of norovirus.

### Case 4

25 weeks of gestation, 740 g, increasing oxygen demand, recurrent severe apnea with bradycardia parallel to the detection of norovirus. Recurrent severe infection during a two month period with constant proof of norovirus shedding. No abdominal symptoms.

### Case 5

32 weeks of gestation,

1. twin, 990 g, distended abdomen, on day 21 vomiting and distended abdomen, parenteral nutrition, proof of norovirus

2. twin, 1900 g, distended abdomen, greenish – mucous stool with visible blood, norovirus suspected but not surely proven

### Case 6

29 weeks of gestation, 1170 g, distended abdomen, proof of norovirus

### Case 7

32 weeks of gestation,

1. triplet, 1780 g, on day 7 vomiting and distended abdomen for five days, proof of norovirus.

2. triplet, 1260 g, on day 8 acute worsening of the general condition, green gastric remainder, increasing oxygen demand, increasing apnea and respiratory insufficiency, then mucous stool with visible blood followed by colitis and coecostomy, capillary leak and multi organ failure, death on day 14. Norovirus not surely proven but proof of rotavirus

3. triplet, 1480 g, on day 11 slight worsening of the general condition with pale marble like skin colour, gastric remainder, distended abdomen, stool with visible blood, no proof of norovirus.

### Case 8

29 weeks of gestation, 1220 g, on day 4 slight gastric remainder with fresh blood, proof of norovirus infection

All different clinical symptoms are summarized in table 1:

**Table 1:** Clinical symptoms of all cases

	Case 1	Case 2	Case 3	Case 4	Case 5.1	Case 5.2	Case 6	Case 7.1	Case 7.2	Case 7.3	Case 8
Respiratory insufficiency	X								X		

Apnea/Tachypnea	X	X		X					X		

Additional oxygen	X	X		X					X		

Sepsis like	X	X									

Anemia	X	X									

Thrombocytopenia		X									

Tachycardia		X									

Gastric Remainders			X						X	X	X

Distended Abdomen		X	X		X	X	X	X		X	

NEC/colitis		X							X		

Vomiting			X		X			X			

Recurrent severe infections				X							

Blood stool						X			X	X	

Blood in Gastric Remainders											X

Skin colour										X	

Death									X		

Proof of Virus											

In stool	X	X	X	X	X	(?)	X	X	(?)		X

In tracheal aspirate	X	X									

## Discussion

Norovirus has not been proven in all our patients but according to the case categories of the German Federal Health Office (Robert – Koch – Institute, Berlin, Germany) norovirus infection can be assumed in an outbreak with similar clinical courses, proof of norovirus and/or with an epidemiological connection [14]. Other similar definitions have been also used in defining norovirus outbreak [15,16]. Also the given sensitivity and specificity of ELISA tests can lead to negative results despite the presence of norovirus [17]. The test used at our laboratory was a commercial ELISA test kit (Ridascreen Norwalk like virus, R-Biopharm, Darmstadt, Germany) [18].

Two recent studies question the validity of enzyme immunoassays (ELISA) in norovirus infection and request RT-PCR instead [12,13]. It seems true that the currently available methods vary greatly in sensitivity, specificity and scope for the detection of norovirus [17]. RT-PCR was not available in our study but in a study by Duizer et al. the sensitivity using ELISA increases with sufficient positive samples [19]. In another study by Rabenau et al. which compared ELISA, PCR and transmission electron microscopy it was shown that all three methods are useful [20]. The discrepancies seen can be explained by the different components each method detects. Even though PCR has the highest sensitivity a negative PCR would not necessarily exclude norovirus infection. Having a source with proven norovirus infection plus the typical clinical course in a mother of one of our hospitalised preterm babies and eight positive samples plus a much better sensitivity in our test kit we therefore see the results of our ELISA being sufficient to confirm norovirus outbreak in our patients.

General clinical symptoms of norovirus infection are described as diarrhea, vomiting, nausea, abdominal cramps, fever and malaise, whereas vomiting occurs more frequently in children and diarrhea more typically in adults [14]. The disease typically lasts one to four days, is self-limited and does not cause chronic infection. Shedding of human calicivirus can last for two weeks and younger children tend to shed for a longer period than older children [21]. Viruses can be detected in stool specimens of some children for a longer period without any signs for illness [22].

Looking at the wide variety of clinical courses in our patients it becomes clear that gastrointestinal problems are the leading symptoms also in neonates (81.8%). According to Kaplan et al. one should always consider norovirus infection in case of explosive vomiting in more than 50% of the patients, acute diarrhea for a period of 12 to 60 hours with an incubation period of 6 to 48 hours [23]. Given these criteria vomiting occurred in only 27% of our patients whereas 63% suffered from distended abdomen. None of our patients suffered from acute diarrhea and apart from abdominal distension only one third showed signs of a lower gastrointestinal tract involvement. A very recent study by Turcios-Ruiz et al. described an outbreak of necrotizing enterocolitis caused by norovirus especially in small premature infants which supports our observation in case 2 and our assumption that case 7.2 suffered also from norovirus infection although the norovirus infection has not been confirmed in this case [24].

During the outbreak all patients of the NICU received a regular screening for norovirus. We found no asymptomatic carriers in our patients.

In general pulmonary symptoms are often the first non specific sign for infection in premature born infants but interestingly these last longer in patients with norovirus infection. Given the immaturity of the immune system of the premature born organism it is surprising that in one case we nearly did not see any signs for infection whereas it is known that the susceptibility of human to norovirus infection is determined by allelic variation in human histo-blood group antigens (HBGA) as described by Huang and others [25]. Proof of norovirus in tracheal aspirate has not been described before (case 1 and 2) and was done in our cases as we initially could not explain the high increase of oxygen demand. Apart from a direct infection of respiratory mucosal cells it might be possible that external contamination could have led to a contamination of respiratory secretions and also microaspiration.

Currently norovirus is divided into 7 genogroups with more than 40 genetic clusters but many aspects of norovirus biology are not well understood [26]. The murine norovirus model system provides the first opportunity to understand the mechanism and pathogenesis of the infection [27]. In this mouse model Karst et al. were able to show that severely immunocompromised mice lacking the signal tranducer and activator of transcription 1 (STAT1) had high levels of virus RNA in all organs examined. Also these mice had histopathological signs of pneumonia and loss of splenic architecture and severe liver inflammation after oral inoculation [27]. Seeing the premature infant as a "naturally" immunocompromised patient this observation might explain the wide variety of clinical courses.

One might only speculate whether a prolonged virus shedding and so related immune reaction may lead to a higher susceptibility to bacterial infection in premature born infants as seen in case 4 or whether it is only expression of an immunocompromised situation with an extended time for recovery.

A couple of problems are pathognomonic for NICUs worldwide: there is rarely a larger space between the incubators. The opening of a heated incubator is similar to an overpressurized chamber and will inevitabely lead to an airborne spread of particles. The frequent visit from parents and relatives and even smaller infants are highly supported to strengthen the bonding between the preterm infant and its family. This all can lead to a persistent and circulating infection especially in a semi-closed community as described earlier [6].

Therefore only the consequent use of different hygienic pattern can lead to elimination of norovirus in such a setting. In our case it was wearing single use coats, gloves and surgical face mask whenever a patient was handled and increased use of norovirus active disinfectants as hand disinfection and on the ward (wiping of floor and surfaces especially around incubators). At the beginning we used a hand disinfectant which was recommend as highly effective on enveloped and non-enveloped viruses although not specifically tested against feline calicivirus (surrogate virus for norovirus) according to the Robert – Koch – Institute [28]. Its pharmaceutically active ingredients contain Ethanol 95.0 g (on 100 g, Sterillium Virugard™, Bode Hamburg/Germany). As we could not stop the outbreak herewith we changed to a new, ethanol reduced hand disinfectant: Manorapid Synergy™ (Antiseptica, Pulheim/Germany). Its pharmaceutically active ingredients contain 10 g 1-Propanol, 57,6 g Ethanol 96% with 0,7% phosphoric acid (on 100 g). This disinfectant produced a log_10 _reduction factor of 2.38 in testing against feline calicivirus compared to other disinfectants with increased ethanol content [29]. For floor disinfection Perform™ 1% (Schülke & Mayr, Norderstedt/Germany) was used which is an active oxygen based highly effective disinfectant containing 45 g Pentakalium bis(peroxymonosulfate)bis(sulfate) and has been tested as being effective against feline calicivirus.

All symptomatic patients underwent strict cohortation and care by dedicated nurses, who were not allowed to care for other patients and to leave that area during work. Relatives were not allowed to get into personal contact with their babies or to peform "kangarooing" as long as we could prove shedding of virus.

With this hygienic management we were able to limit the disease and completely terminate it after two months (case 4).

Finally, although not in our case, it can be necessary to close a ward completely until the virus is eradicated. This "worst case scenario" leads to a loss of proceeds which was calculated by Lopman et al. as 1,01 million $ per 1000 hospitals beds [30].

## Competing interests

The authors declare that they have no competing interests.

## Authors' contributions

SA planned and carried out the study, collected data and wrote the manuscript. AK planned and carried out all hygienic measures and revised the manuscript critically. DO participated substantially in the design of the study and data collection. KZ carried out the ELISA testing. CF controlled the study and revised the manuscript critically for important intellectual content

All authors read and approved the final manuscript to be submitted to BMC Research Notes.
